# Pandemic March: 2019 Coronavirus Disease’s First Wave Circumnavigates the Globe

**DOI:** 10.1017/dmp.2020.103

**Published:** 2020-04-16

**Authors:** James M. Shultz, Alanna Perlin, Russell Gary Saltzman, Zelde Espinel, Sandro Galea

**Affiliations:** Center for Disaster and Extreme Event Preparedness (DEEP Center), Miami, Florida; Department of Public Health Sciences, University of Miami, Miami, Florida; University of Miami Leonard M. Miller School of Medicine, Miami, Florida; Sylvester Comprehensive Cancer Center, Department of Psychiatry and Behavioral Sciences, Miami, Florida; School of Public Health, Boston University, Boston, Massachusetts

**Keywords:** case-fatality rate, coronavirus, COVID-19, epidemiology, global spread

## Abstract

**Objective:**

March 2020 was a pivotal month for the worldwide geographic and numeric expansion of the first wave of Coronavirus Disease 2019 (COVID-19). We examined the major storylines that depicted this explosive spread of COVID-19 around the globe.

**Methods:**

A detailed review of World Health Organization (WHO) situation reports, surveillance summaries, and online resources allowed us to quantify the increases in cases and deaths by region and by country throughout the month of March 2020.

**Results:**

During March, COVID-19 was officially declared by the WHO to be a pandemic. COVID-19 emerged from a focalized outbreak in the Western Pacific Region and rapidly proliferated across all continents worldwide. Globally, cumulative numbers of confirmed cases increased by a factor of nine throughout the month. During the entire month, cases rose exponentially throughout Europe. Starting in mid-March, confirmed cases accelerated coast-to-coast throughout the United States and, on March 26, the United States surpassed all other nations to rank first in numbers of cases. COVID-19 mortality lagged several weeks behind but by month’s end, death tolls were also rising exponentially.

**Conclusion:**

March 2020 was a consequential month when the COVID-19 pandemic wrapped completely around the planet, with outbreaks erupting in most nations worldwide.

The month of March 2020 was pivotal for the worldwide progression of coronavirus disease 2019 (COVID-19). COVID-19 was previously designated by the World Health Organization (WHO) as a Public Health Emergency of International Concern (PHEIC) on January 30, 2020. However, it was on March 11, 2020, when WHO Director General, Dr Tedros Adhanom Ghebreyesus, declared the outbreak to be a pandemic. March witnessed the total global wraparound of this highly transmissible disease.

By the month’s end, the worldwide geographic distribution of cases had been dramatically reshaped. COVID-19’s reach extended across 6 continents and all United Nations regions, with cases reported in 205 countries and territories. Although the tallies of confirmed cases and deaths will be eclipsed, and in fact dwarfed, in future months, it was during March 2020 that COVID-19 demonstrated its remarkable capacity to circumnavigate the planet. COVID-19 became a worldwide threat with breathtaking speed.

We examine patterns of confirmed cases globally, by United Nations regions and by country, drawing from WHO daily situation reports throughout the month of March 2020, with particular focus on the reports issued on March 1 and April 1, 2020.^1,2^

## CONFIRMED CASES OF COVID-19

Dating from the index case in Wuhan, China, reported in late December 2019, through the end of February 2020, diagnosed cases of COVID-19 were concentrated in the Western Pacific. On the final day (29th) in February – Leap Day, 2020 – the WHO case count stood at 87 137, with 79 968 cases (91.8%) in China, 3736 (4.3%) in South Korea, and 411 (0.4%) throughout the remainder of the Western Pacific Region – with 239 of these reported in Japan (Table 1). Only 3022 cases (3.5%) resided outside of the Western Pacific, including 1128 in Italy and 593 in Iran. Another 705 essentially “stateless” passengers remained sequestered on the Diamond Princess cruise ship. Apart from China, South Korea, Japan, Singapore, Italy, Iran, and France, no other country had reached 100 reported cases. Only China, South Korea, and Italy had more than 1000 reported cases. The entire Western Hemisphere – the WHO “Americas” region – had only 86 reported cases, with 62 in the United States and 19 in Canada.

**TABLE 1:** Numbers of Countries Reporting COVID-19 Cases, and Numbers of Cumulative Cases Reported by United Nations Region on February 29, 2020, and March 31, 2020

	Countries Reporting COVID-19			COVID-19 Cumulative Cases		
United Nations Region	February 29, 2020	March 31, 2020	X Times Increase	February 29, 2020	March 31, 2020	X Times Increase
China	1	1	NA	79 968	82 631	1.0
Other Western Pacific	9	18	2.0	4147	23 791	5.7
European Region	27	60	2.2	1457	464 212	318.6
Region of the Americas	5	51	10.2	86	188 751	2194.8
Eastern Mediterranean Region	11	21	1.9	725	54 281	74.9
Southeast Asian Region	4	10	2.5	47	5175	110.1
African Region	2	44	22.0	2	4073	2036.5
Diamond Princess	NA	NA	NA	705	712	1.0
**Totals**	**59**	**205**	**3.5**	**87 137**	**823 626**	**9.5**

In terms of known, confirmed cases, whether plotted on a map or depicted on an epidemic curve, COVID-19 appeared as a regional outbreak constrained primarily to the Western Pacific. However, as a faint but ominous sign of what March would bring, a total of 59 countries worldwide had reported cases to the WHO by February 29, including 50 countries outside of the Western Pacific Region. Yet among these 59 nations, 40 reported 4 cases or fewer.

During March 2020, all changed. Globally, confirmed case counts vaulted upward exponentially to 823 626 cases on March 31, 2020, representing more than a 9-fold increase in cases worldwide. Beyond the cumulative numbers, the global distribution of cases was fundamentally altered within a single month.

Three storylines emerged. First, extending throughout the entire month of March, COVID-19 engulfed the European Region, which would end the month with more cases than any other United Nations region. Second, continuously gaining momentum, COVID-19 swept generally west to east, and north to south throughout the continental United States, which would end the month with far more cases than any other nation. Third, by the end of March, as the COVID-19 pandemic spread broadly, 205 nation states and territories had reported cases - 3.5 times more than the 59 states and entities reporting cases as of February 29, 2020.

### Storyline 1: European Region

On February 29, The European Region had just 1457 COVID-19 cases, representing 1.7% of the global total. Fully 1128 (77.4%) of the European cases were reported in Italy. As a harbinger of things to come, 27 European countries had reported cases, yet 15 European nations had 3 cases or fewer.

**FIGURE 1:**
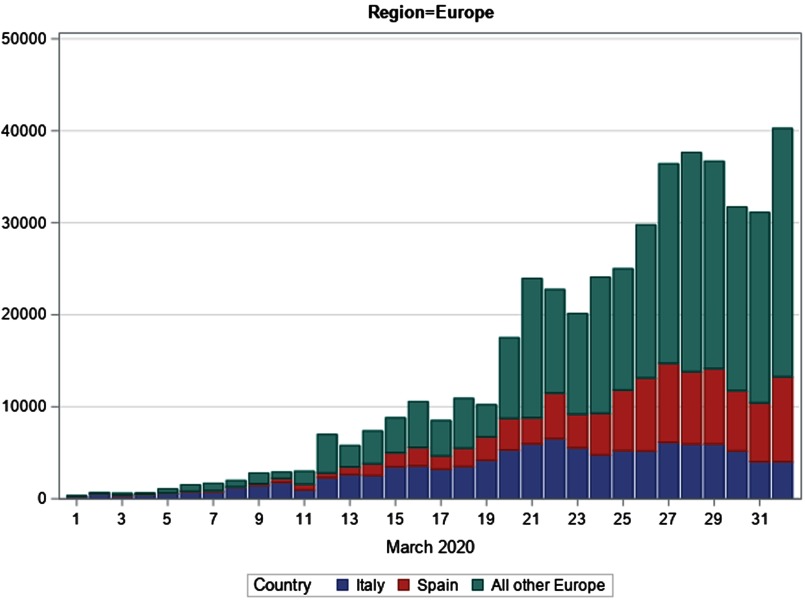
European Region: Daily confirmed cases of COVID-19, March 1-April 1, 2020

During March, the European Region cases would increase to 464 212 (56.4% of total global cases), with cumulative cases rising by a factor of 319 over the month (Figure 1). The number of European Region nations reporting cases more than doubled from 27 to 60 countries and territories. Italy exceeded 100 000 cases. Italy and Spain both surpassed the numbers of cumulative cases reported in China. By the month’s end, 24 European nations and territories had more than 1000 reported cases

### Storyline 2: United States

On February 29 and March 1, 2020, the United States had 62 confirmed cases of COVID-19. Two weeks later, on March 15, the US case count had increased 27-fold to 1678, having doubled almost 5 times. By March 31, cases had doubled 6 more times to 163 199.

**FIGURE 2:**
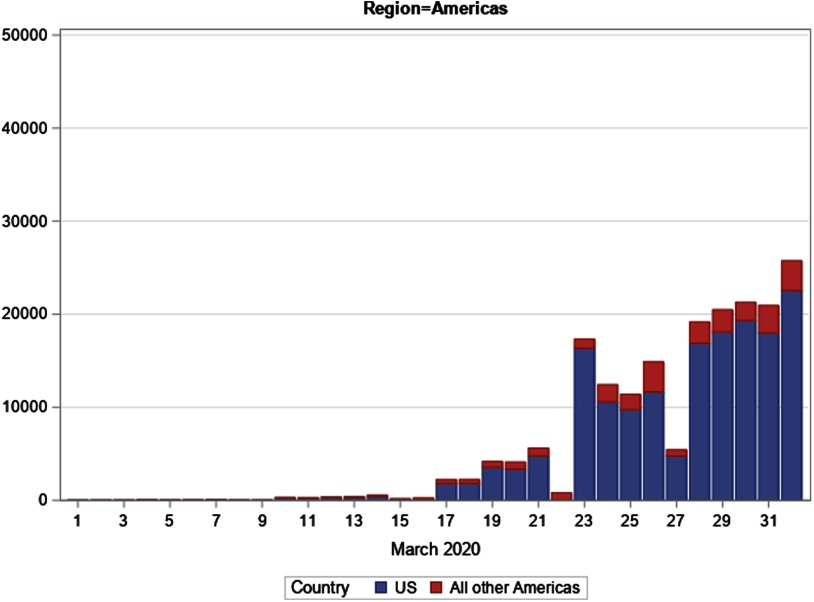
Region of the Americas: Daily confirmed cases of COVID-19, March 1-April 1, 2020

On March 24, 2020, the WHO predicted that the United States was becoming the new global epicenter for COVID-19. Indeed, just 2 days later, rapidly rising case counts catapulted the United States into first place. During the month of March, the United States leapfrogged over multiple nations that initially had more reported cases, including Japan, South Korea, France, Iran, Germany, Spain, and, finally, China and Italy on March 26. US case counts continued to accelerate for the remainder of March (Figure 2). During the final week, the United States was contributing about 30% of global new cases each day, far in excess of any other country.

At month’s end, the United States stood alone as the number 1 nation in numbers of confirmed COVID-19 cases. Just 5 days after tying and passing China’s cumulative case total, the United States ended the month of March with double the number of confirmed cases in China. During March 2020, US cases increased by a factor of 2632.

Geographically, the spread of COVID-19 across the United States is occurring unevenly, and hotspots may appear unexpectedly. In early March, King County, Washington, the site of the nation’s first major outbreak, witnessed a rapid escalation of cases. In Westchester County, New York, including the town of New Rochelle, there was a steep upsurge in cases. During the final 10 days of March, New York City, along with the State of New York, became the US COVID-19 epicenter. On March 31, half of the cases in the United States were in New York State (40%) and neighboring New Jersey (10%), with the highest concentration in the New York City metropolitan area (Table 2).

**TABLE 2:** Top 10 US States in Numbers of Cumulative COVID-19 Cases and Reported Deaths on March 31, 2020

State	Cumulative Cases	Deaths	Case-Fatality Rate
**New York**	83 889	1941	2.3%
**New Jersey**	22 255	355	1.6%
**California**	9599	206	2.1%
**Florida**	7765	101	1.3%
**Michigan**	7630	264	3.5%
**Massachusetts**	6620	89	1.3%
**Louisiana**	6424	273	4.2%
**Illinois**	5994	146	2.4%
**Pennsylvania**	5805	74	1.3%
**Washington State**	5378	248	4.6%

At the end of March, 8 additional states were clustered in the 5000–9000 case range: California, Michigan, Florida, Massachusetts, Illinois, Washington, Louisiana, and Pennsylvania. Outbreaks were flaring in multiple urban centers within these states, including Los Angeles, Detroit, Chicago, and New Orleans. South Florida cases were rising steeply, with speculation that sources of infection included visits by students populating Florida beaches and clubs during spring break, residents from the epicenters in the northeast fleeing to their second homes in Florida, and cruise ship passengers and personnel.

### Storyline 3: Worldwide Dispersion

The COVID-19 pandemic extended into 205 nation states and territories by the end of March compared with 59 entities reporting cases at the end of February. During that interval, the number of countries/territories with more than 100 cases had grown by a factor of 16, from 7 nations to 115. Two regions, in particular, witnessed monumental increases in nations reporting cases with the region of the Americas experiencing a 10-fold rise and the African Region experiencing a 22-fold increase.

As noted, Europe accounted for 56.4% of total global cases reported at the end of March, with case counts rising by a factor of 319. Yet this number, startling in its own right, was greatly exceeded by the more than 2000-fold increase in confirmed case numbers in both the region of the Americas and the African Region.

## COVID-19 DEATHS AND CASE-FATALITY RATES

### Global Picture

At the beginning of March, China had recorded 2873 deaths, and just 104 deaths were reported worldwide from countries other than China. By the end of March, the global death toll had risen to 40 598. Among the 37 621 deaths occurring during March, only 448 deaths occurred inside China. Cumulative non-China deaths had increased by a factor of 358. The overall global case-fatality rate (CFR) was 4.9% but with remarkable disparities by region and by country within a region (Table 3).

**TABLE 3:** Numbers of Cumulative Cases and Fatalities Reported by United Nations Region on February 29, 2020, and March 31, 2020, and Computed Case-Fatality Rate

	February 29, 2020			March 31, 2020		
United Nations Region	Cases	Fatalities	Case-Fatality Rate	Cases	Fatalities	Case-Fatality Rate
China	79 968	2873	**3.6%**	82 631	3321	**4.0%**
Other Western Pacific	4147	24	**5.8%**	23 791	380	**1.6%**
European Region	1457	31	**2.1%**	464 212	30 089	**6.5%**
Region of the Americas	86	0	**0.0%**	188 751	3400	**1.8%**
Eastern Mediterranean Region	725	43	**5.9%**	54 281	3115	**5.7%**
Southeast Asian Region	47	0	**0.0%**	5175	195	**3.8%**
African Region	2	0	**0.0%**	4073	91	**2.2%**
Diamond Princess	705	6	**0.9%**	712	7	**1.0%**
**Totals**	**87 137**	**2977**	**3.4%**	**823 626**	**40 598**	**4.9%**

#### European Region

On March 1, the European Region recorded 1457 cases and 31 deaths, with a CFR of 2.1%. By the month’s end, those numbers had incremented to 464 212 cases and 30 089 deaths with a CFR of 6.5%. Cumulative deaths increased by a factor of 970. Several countries in the European Region had extremely high CFRs that were rising steadily over the month. On March 31, the CFR for Italy was 11.7% and for Spain, 8.7%.

#### United States

The first US death was announced on February 29, 2020, but it did not appear on the WHO situation reports until March 2, 2020. US COVID-19 deaths increased slowly during the first half of March, but, during the final week, death tolls were setting new highs for each day. On March 31, US COVID-19 deaths had risen to 2850 among 163 199 cases for a CFR of 1.7%. At the month’s end, the US CFR was rising and had not yet stabilized. Very simply, the disease course for severe and potentially fatal COVID-19 often extends over weeks. During the month of March, cases were surging upward during the final 2 weeks, which was insufficient time for many with positive test results to progress to severe disease. Patients had not had sufficient time to die from severe COVID-19 illness.

## CONCLUSION

The first wave of COVID-19 cases is on the move, rampantly extending its reach throughout the globe and erupting in new hotspots worldwide. April 2020 will bring many more cases and deaths. In terms of what to expect beyond the explosive growth of the COVID-19 pandemic in March, much will depend upon the ability to conduct disease surveillance, and this will depend upon widespread availability of tests for COVID-19, case identification, contact tracing, and isolation of persons who are infectious. On a population basis, the ability to enact and achieve adherence to mitigation and suppression measures, such as social distancing, will modulate the extent of viral spread, disease, and death.^3^ Disease modeling is being conducted worldwide. Two models that have influenced policy-making regarding mitigation measures for the United States have been conducted by Imperial College London^3^ and the University of Washington’s Institute for Health Metrics and Evaluation.^4,5^

We have chosen to selectively focus on March 2020 as the month when COVID-19 burst out of the Western Pacific Region and moved swiftly and devastatingly around the globe. March 2020 was the “breakout” month when COVID-19 transformed into its full pandemic presentation, appearing seemingly everywhere in a matter of weeks.
